# Verification of nonwords: The baseword frequency effect in children’s pseudohomophone reading

**DOI:** 10.3758/s13423-017-1424-3

**Published:** 2018-01-12

**Authors:** Simon P. Tiffin-Richards, Sascha Schroeder

**Affiliations:** MPRG REaD (Reading Education and Development), Max Planck Institute for Human Development, Berlin, Germany

**Keywords:** Visual word recognition, Cognitive development, Orthography, Phonology

## Abstract

In this study, we investigated the baseword frequency effect in children and its implications for models of visual word recognition. The baseword frequency effect reflects the finding that response latencies in the lexical decision task to nonwords derived from high-frequency basewords (e.g., GREAN derived from GREEN) are shorter than for those derived from low-frequency basewords (e.g., SLEAT derived from SLEET). Importantly, the baseword frequency effect presents a challenge to current activation-based models of visual word recognition. One explanation for this effect is that the orthographic representations of high-frequency basewords are easier to access. This allows a quick progression to a verification stage in which the exact spelling of a stimulus is checked, upon which the lexicality decision is then based. The main goal of this study was to investigate whether such a verification mechanism is specifically modulated by the quality of the orthographic lexicon. We tested whether the baseword frequency effect was evident in children’s lexical decisions to pseudohomophones (PsH) and whether verification accuracy varied as a function of children’s orthographic knowledge. The baseword frequency effect in response latency was observed in both German-speaking adults and children. Children’s spelling skills significantly influenced the accuracy of the verification stage in their responses to PsH. These findings imply that verification is an integral part of word reading and thus should be included in computational models of visual word recognition.

**Electronic supplementary material:**

The online version of this article (10.3758/s13423-017-1424-3) contains supplementary material, which is available to authorized users.

The study of lexical processing has provided an extensive understanding of word properties that influence the speed and accuracy of visual word recognition (e.g., number of letters, printed frequency, number of orthographic neighbors) and informed the development of computational models of word recognition (Balota et al., 2004). However, the processing of nonwords, for instance in the lexical decision task in which words must be distinguished from nonwords (LDT; Rubenstein et al., 1970), is less well understood (Yap et al., 2015). Studies have shown that nonwords that sound like real words (pseudohomophones) are harder to reject in LDT than nonwords that do not (Frost, 1998; Ziegler et al., 2001). This suggests that pseudohomophones (PsH) are decoded, activating the corresponding entry in the phonological lexicon, which creates a conflict because no orthographic match is available, resulting in higher cognitive load, longer response times, and higher error rates compared with spelling controls (Briesemeister et al., 2009). The baseword frequency effect (van Orden et al., 1992) reflects the finding that LDT response latencies to nonwords derived from high-frequency basewords (e.g., GREAN from GREEN) are shorter than for those derived from low-frequency basewords (e.g., SLEAT from SLEET), similar to the word frequency effect (Balota et al., 2004). This has been replicated in adult readers (Yap et al., 2015; Ziegler et al., 2001), although with some inconsistency (Perea et al., 2005). In this study, we investigated whether children show the baseword frequency effect in LDT and whether it is influenced by their spelling skill.

Importantly, the baseword frequency effect challenges activation-based models of visual word recognition. For example, the dual-route model is able to simulate LDT responses (DRC, Coltheart et al., 2001) using a multiple read-out mechanism (M-ROM, Grainger & Jacobs, 1996) in which a YES response is produced when a word reaches a threshold level of lexical activation. If the activation threshold is not reached before a deadline, a NO response is generated. The deadline is variable depending on the level of global activation (Grainger & Jacobs, 1996), where high global activation extends the deadline, delaying the lexical decision. Nonwords that share orthographic or phonological features with words stored in the mental lexicon produce global activation, delaying NO responses. Activation-based models predict that nonwords with high baseword frequency result in higher global activation and longer response times than nonwords with low baseword frequency, which is at odds with empirical findings. Similarly, more recent diffusion models (Ratcliff et al., 2004) assume that the decision process in LDT is based on accumulated evidence indicating the presence of a word compared with the evidence for a nonword. However, what constitutes evidence for a nonword is not entirely clear and NO decisions may be based on the difference between the evidence for a word and a baseline level of activation (Dufau et al., 2012). Nonwords with high-frequency basewords would thus be more difficult to reject, which again conflicts with the baseword frequency effect.

A mechanism proposed to account for the baseword frequency effect involves a verification stage in addition to lexical activation (Bergman & Wimmer, 2008; Paap et al., 1982; Ziegler et al., 2001). This mechanism can be reconstructed in more recent versions of the dual-route model of reading, where location-specific orthographic codes (Grainger & Ziegler, 2011) are mapped onto phonemes to activate the phonological baseword representation. Consequently, nonwords with high frequency basewords gain faster access to their phonological representation. Verification in LDT then involves the comparison of the exact letter identities and positions with the orthographic baseword representation through the fine-grained orthographic route. If deviations are detected, a NO response is generated.

The verification mechanism in word recognition can be assumed to be highly automatized and accurate in skilled adult reading, as high accuracy rates in LDT studies indicate. However, primary school children and dyslexic adolescents are far less accurate in rejecting nonwords in LDT (Bergmann & Wimmer, 2008; Richter et al., 2013). Errors may occur when verification via the fine-grained orthographic route fails to detect differences between a presented nonword and its baseword and an incorrect YES response to the PsH is generated through phonological activation. The quality of the baseword’s orthographic representation is likely to influence the accuracy of verification. In this case, quality refers to the specification of letter identity and position information stored as part of the orthographic representation, which is necessary to detect deviations between nonwords and their basewords via the fine-grained orthographic route (Grainger & Ziegler, 2011). As orthographic representations are consolidated through repeated exposure (Stanovich & West, 1989), their specificity will likely vary considerably in beginning readers. It follows that children’s verification accuracy should depend on the quality of their orthographic representations which are not as highly defined as those of skilled adult readers. In line with this assumption, previous studies have shown that errors in detecting PsH as nonwords decrease across reading development and are strongly related to participants’ reading age (Grainger et al., 2012). It is unclear, however, whether the effect is related to the efficiency of the activation or the verification mechanism (or both). In addition, we do not know whether children’s decreasing error rates in detecting PsH are related to the development of their orthographic lexicon.

To investigate the link between the verification mechanism and the orthographic lexicon, we conducted a LDT with a large sample of fourth-grade children and a group of adults. The nonword stimuli used were PsH, which shared phonology and all but one letter with their baseword (e.g., HANT from HAND, pronounced /hant/, engl. hand). Importantly, the frequency of the basewords that were used to generate the PsH was manipulated experimentally. This allowed us to investigate the effects of baseword frequency on responses to the PsH. We tested two main hypotheses.

First, assuming the speed of accessing representations and the accuracy in evaluating their lexicality rely on two different underlying processes, namely activation and verification, we expect a baseword frequency effect in children’s response times, despite their poor verification accuracy. This would suggest that children, similar to adults, are able to activate high-frequency basewords faster than low-frequency basewords, independently of the accuracy of the final verification stage. In addition, we assumed that if the accuracy of the verification mechanism depends on the quality of children’s orthographic representations, it should be related to their spelling skill. Our second hypothesis was thus that the baseword frequency effect would be greater for children with good spelling skills, as they have more stable orthographic representations for high frequency words.

## Method

### Participants

We recruited 212 fourth-grade children in Berlin with the written consent of their parents. Eight performed below chance level (50%) and six had not started learning German by the age of six years, leaving an effective sample size of 198 (103 girls, age *M* = 9 years, 6.7 months, *SD* = 5.7 months). An additional 30 adults (18 women, age *M* = 24, *SD* = 3 years) were recruited. One adult was excluded as their first language was not German.

#### Lexical decision task

In the LDT, participants were presented a word or PsH on the screen and instructed to indicate if it was spelled correctly. Each trial was preceded by a 500-ms fixation cross after which the next trial was displayed until the participant made a response by pressing a YES (right) or NO (left) key. Two practice items were followed by 80 experimental trials in random order.

The stimuli comprised 160 baseword nouns, verbs, and adjectives, of which 80 had a high lemma frequency (*M* = 83 occurrences per million in the childLex corpus; Schroeder et al., 2015) and 80 had a low lemma frequency (*M* = 5 occurrences per million). To derive PsH a single letter was exchanged in each baseword to obtain a phonologically identical nonword (e.g., Träne, Trene, pronounced /tʀε:nə/, engl. tear). The PsH and basewords had equivalent bigram frequencies, were of equal character length, and had the same number of orthographic neighbors, *t*s < 2. The resulting pool of 160 basewords and 160 corresponding PsH was split into two lists, each containing 20 high-frequency and 20 low-frequency words, as well as 20 high baseword frequency PsH and 20 low baseword frequency PsH. There were no differences in average word frequency, baseword frequency, bigram frequency, character length, or neighborhood size between lists, *t*s < 2.

#### Spelling skill

The Hamburger Schreib-Probe (HSP 1-9, May, Vieluf & Malitzky, 2002) measured children’s spelling skill and consisted of 16 single words and five sentences comprising 26 words. The correct spellings were aggregated to a single score (*M* = 25.33, range = 6-38, *SD* = 7.54).

### Procedure

Children completed the spelling assessment in their classroom. In a second, computer-based session, children completed the LDT on 15-inch laptops in groups of 10 to 20. Participants were randomly assigned to one of the two LDT lists and response accuracy and latency were recorded. Each child received a small gift after the second session. Adults took part in a single group session of 5 to 10 participants in which they completed the LDT and received €10 in compensation.

### Analysis

The observed mean response latency and accuracy for words and nonwords for children and adults are displayed in Table 1. Response accuracy and log-transformed response latency were analyzed using linear mixed-effects models in R (R Core Team 2015) with the lme4 package using the glmer function for response accuracy and lmer function for response latency (Bates et al., 2014). For the analysis of response latency to nonwords, only correct responses above 300 ms and below 10,000 ms were included. Responses 2.5 *SD* above the mean latency for each item and participant were discarded, excluding less than 2% of data. For the analysis of response accuracy to nonwords, only responses above 300 ms and below 10,000 ms were included. Means and standard errors for conditions and contrasts were estimated using cell-mean coding. Note that all reported effect sizes were back-transformed from their log(latency) and logit(accuracy) estimated fixed effects.

**Table 1:** Observed mean response latency and accuracy to nonwords with high and low baseword frequency and high and low frequency words with standard errors for children and adults

Frequency	Nonwords		Words^a^	
	Latency	Accuracy	Latency	Accuracy
*Children*				
High	2031 (27)	0.53 (.008)	1466 (17)	0.92 (.004)
Low	2145 (30)	0.51 (.008)	1750 (23)	0.73 (.007)
*Adults*				
High	733 (9)	0.93 (.011)	621 (8)	0.99 (.003)
Low	768 (11)	0.90 (.013)	702 (10)	0.93 (.011)

^a^The observed mean response latencies and accuracies for words are included for comparison

## Results

In a first step, we investigated whether adults and children showed baseword frequency effects. To this end, separate analyses were run on adults’ and children’s nonword responses with frequency as a categorical, effect-coded factor.

Adults showed a strong baseword frequency effect in response latency, *F*(1,146.25) = 11.76, *p* < 0.001. Responses for nonwords derived from high-frequency words were faster than responses for nonwords derived from low-frequency words. By contrast, the baseword frequency effect for response accuracy was not significant, χ^2^ (1) < 1, *p* = 0.351. Adults’ response accuracy to nonwords was close to ceiling performance (Table 1).

Children also showed a baseword frequency effect in response latency, *F*(1, 156.63) = 4.36, *p* = 0.038, which was considerably smaller than the one for adults. Responses for nonwords derived from high-frequency words were faster than responses for nonwords derived from low-frequency words. Similar to adults, children did not show a baseword frequency effect for response accuracy, χ^2^ (1) < 1, *p* = 0.392. However, response accuracy for nonwords with high baseword frequency was significantly above chance, *t*(197) = 2.07, *p* = 0.039, while it was not for nonwords with low baseword frequency, *t*(197) = 0.37, *p* = 0.714.

In summary, the response patterns for adults and children were rather similar overall with clear baseword frequency effects for response latency but not response accuracy. Children’s performance level was generally much lower than for adults. In a next step, we investigated how children’s performance on nonwords and the size of the baseword frequency effect was related to their spelling skill. To this end, children’s spelling scores were centered and included as a continuous variable in the model.

For response latency, there was a significant main effect of children’s spelling skill. Children scoring 1 *SD* above the mean had average response latencies to nonwords, which were 380 ms faster than those of children scoring 1 *SD* below the mean. The baseword frequency effect was not moderated by spelling ability.

**Fig. 1:**
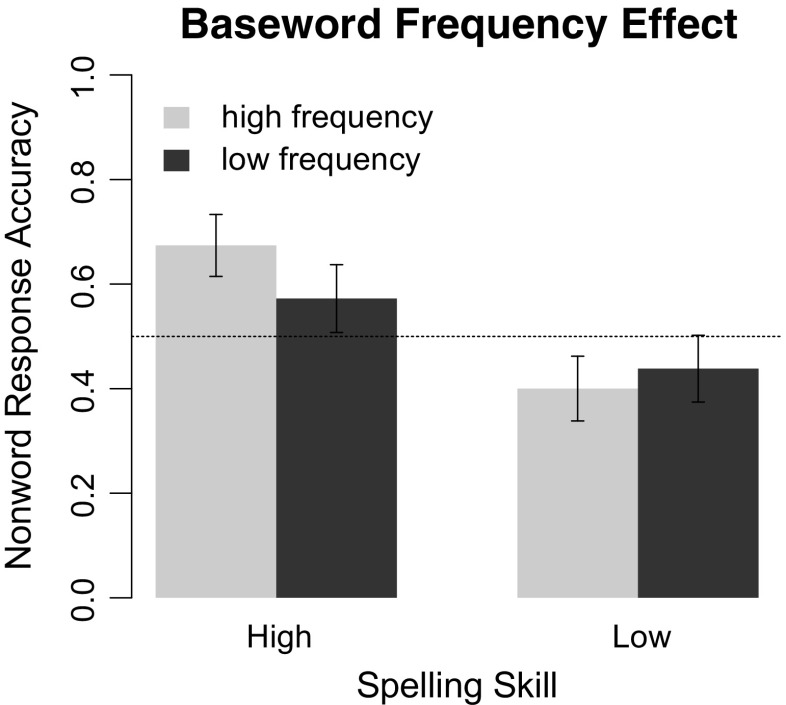
Baseword frequency effects in model means of response accuracy to nonwords for high- and low-skilled spellers

For response accuracy, we observed a different pattern (Table 2). First, there was a strong main effect of children’s spelling skill: Children scoring 1 *SD* above the mean rejected nonwords 21% more accurately than children scoring 1 *SD* below the mean. Furthermore, children’s spelling skills also moderated the baseword frequency effect. This interaction is displayed in Fig. 1. For children scoring 1 *SD* above the mean, responses to nonwords derived from high-frequency words were 10.4% more accurate than responses to nonwords derived from low-frequency words, *t* = 2.83, *p* = 0.004. For children scoring 1 *SD* below the mean, by contrast, responses to nonwords derived from high- and low-frequency words did not significantly differ from each other, *t* = 1.05, *p* = 0.293. As the dashed line in Fig. 1 illustrates, only high-skilled spellers performed above chance level in rejecting PsH.

**Table 2:** Omnibus ANOVA results for the effects of baseword frequency and interactions with spelling skill on response accuracy and latency to nonwords

Factor	Accuracy			Latency		
	χ^2^	*df*	*p* value	*F*	*df*	*p* value
Intercept	<1	1	0.319	78588	1	<0.001
Spelling	53	1	<0.001	16	1	0.041
Frequency	<1	1	0.339	4	1	<0.001
Frequency × spelling	32	1	<0.001	1	1	0.358

These results indicate that good spellers were both faster and more accurate in their responses to nonwords than poor spellers. Most importantly, good spellers showed a greater baseword frequency effect in their response accuracy to nonwords than poor spellers. This suggests that good spellers had an advantage in verifying the spelling of PsH with high frequency basewords due to their higher specification of high frequency orthographic representations.

## Discussion

This investigation replicates previous findings that readers’ performance in responding to nonwords in the LDT is strongly related to their reading level (Grainger et al., 2012). At the same time, it extends previous studies in two important ways. First, our data demonstrate that children show a baseword frequency effect in response latency, indicating that basewords are routinely identified during PsH processing. Nonwords with high-frequency basewords elicit a high degree of lexical activation, resulting in fast retrieval of their basewords, which are then verified using position-specific orthographic codes (Grainger & Ziegler, 2011).

Second, we found a strong effect of children’s spelling skill on response accuracy. This demonstrates that orthographic knowledge has a substantial influence on the accuracy of the verification process. Only children with high-quality orthographic representations were able to reject the PsH above chance level and showed reliable baseword frequency effects. High-skilled spellers had a distinct advantage in rejecting nonwords with high baseword frequency, presumably because they have consolidated more letter identity and position-specific information of high-frequency words in their orthographic lexicon. This supports the assumption that the verification mechanism draws on frequency sensitive orthographic representations and relies on their specificity to detect deviations when ascertaining the lexicality of letter-strings.

Generally, our results replicate the findings of van Orden et al. (1992) and further generalize their findings to children, supporting an activation-verification account of visual word recognition in both adult and beginning readers. The significance of these findings is that activation and verification processes can be dissociated and that beginning readers with poor orthographic representations are not yet able to utilize frequency sensitive information to facilitate verification.

We interpret our findings as evidence that the quality of the entries in the orthographic lexicon drives the accuracy of the verification process. However, as our spelling assessment was an accuracy-based measure, we could not directly compare effects of spelling skill and other speed-based reading measures. Future studies may want to include either comparable speed or accuracy-based measures of both spelling and reading comprehension to test whether reading comprehension and spelling skill selectively influence activation and verification processes, respectively, as the dual route model of orthographic processing would imply (Grainger & Ziegler, 2011).

It is important to note that we presented only PsH nonwords, whereas other studies have mixed PsH with spelling controls (Ziegler et al., 2001; Bergman & Wimmer, 2008). Verification for PsH has been described as a matching process between phonological and orthographic representations in the mental lexicon. Because PsH are identical to the phonological representation of their baseword but differ from their orthographic representation, the size of this mismatch may drive the ease with which they can be detected as nonwords. PsH with high-frequency basewords elicit higher activation of the phonological representation of their baseword, leading to a greater mismatch and facilitation of the verification process (Ziegler et al., 2001). According to Balota and Chumbley (1984), words and nonwords form two distributions along a scale of familiarity-meaningfulness, where the mean value for words is higher than for nonwords. High-frequency words are located on the upper end of the word familiarity distribution and low-frequency words on the lower end. Nonwords have a similar distribution, with word-like nonwords on the upper end. The two distributions overlap at the point where nonwords are highly word-like and words have very low frequency. In this region, precise orthographic information is necessary to determine lexicality accurately. It is therefore likely that using only PsH in this study led to a high overlap in the word and nonword information distributions, increasing the difficulty of the task. However, what the high error rate emphatically demonstrated is that children showed a baseword frequency effect in response latency to PsH, even when their responses were highly inaccurate. This is striking evidence for the dissociation of the activation and verification processes, because fast activation does not guarantee correct verification.

A general assumption of models of visual word recognition is that different processing routes are necessary for words and nonwords (Coltheart et al., 2001), which theoretically requires their distinction at an early stage of processing. The activation-verification approach has the attractive property of being able to assume the same process for words and word-like nonwords, with the distinction being made at the late verification stage. Any letter-string resembling an entry in the mental lexicon elicits lexical activation. The frequency of the activated representations influences the speed of access and is reflected in response latency to both words and nonwords. The following verification stage is mainly relevant in tasks that require exact, fine-grained orthographic information, such as error detection in proofreading and nonword rejection in LDT. Verification is reflected in the accuracy of responses, which may account both for errors in rejecting nonwords and accepting words with underspecified orthographic representations. However, as Ziegler et al. (2001) point out, the presence of a verification mechanism does not necessarily require the separation of activation and verification into two processing stages. The verification process also may be seen as an automatic final aspect of activation or top-down feedback from the activated orthographic representation to the bottom-up evidence for the presence of a word (Ziegler et al., 2001). If verification is seen as a generic aspect of visual word recognition, YES responses to words may be adversely affected when a presented word is checked against an imprecise orthographic representation, resulting in prolonged decision times and increased response errors.

In summary, we replicated the baseword frequency effect in adults and extended it to children, showing that facilitated access to high-frequency representations is to some extent distinct from the accuracy of verification. We further demonstrated individual differences in the expression of the baseword frequency effect in response accuracy, depending on the quality of beginning reader’s orthographic lexicon. The addition of an integral mechanism to computational models of visual word recognition, which draws on orthographic representations to verify the spelling of words, thus appears warranted and particularly relevant when studying young and low-skilled readers.

### Author note

This research was funded by a grant from the German Ministry of Education and Research (BMBF, Förderkennzeichen 01LSA1505A).

## Electronic supplementary material

ESM 1 (DOCX 21 kb)
