# Supplementary material for: Verification of nonwords: The baseword frequency effect in children’s pseudohomophone reading
Source: Psychon Bull Rev. 2018 Jan 12;25(6):2289–94. doi: 10.3758/s13423-017-1424-3 (PMC6267514; doi:10.3758/s13423-017-1424-3)
Supplement: Supplementary file 1 — (DOCX 21 kb) [file 13423_2017_1424_MOESM1_ESM.docx]

**Supplementary Materials: Item List**

Table 3

*Orthographic Decision Items: High Frequency Basewords and Pseudohomophones (PsH)*

| Baseword | PsH | Baseword | PsH |  | Baseword | PsH | Baseword | PsH |
| --- | --- | --- | --- | --- | --- | --- | --- | --- |
| Bär | Ber | Holz | Hollz |  | Ton | Tohn | Gold | Golt |
| Fäuste | Feuste | Wolf | Wollf |  | Dame | Dahme | Grund | Grunt |
| Träne | Trene | Wolke | Wollke |  | Name | Nahme | Kleid | Kleit |
| Träume | Treume | Schatz | Schattz |  | Atem | Ahtem | Kobold | Kobolt |
| nähern | nehern | Spitze | Spittze |  | Kater | Kahter | Land | Lant |
| Gespräch | Gesprech | Kraft | Krafft |  | Pirat | Piraht | Mond | Mont |
| Geräusch | Gereusch | Papier | Pappier |  | Monat | Monaht | Schild | Schilt |
| ändern | endern | Gestalt | Gestallt |  | Brot | Broht | Wind | Wint |
| zählen | zehlen | Vogel | Vogell |  | Pfote | Pfohte | Sand | Sant |
| ärgern | ergern | Insel | Insell |  | Tor | Tohr | Strand | Strant |
| Fell | Fäll | Flamme | Flame |  | Gefahr | Gefar | Ast | Asd |
| fremd | främd | Nummer | Numer |  | strahlen | stralen | Art | Ard |
| gelb | gälb | Sommer | Somer |  | Wahrheit‡ | Warheit‡ | Brust | Brusd |
| Berg | Bärg | Bitte | Bite |  | Lehrer | Lerer | Gast | Gasd |
| Decke | Däcke | Lippe | Lipe |  | stehlen | stelen | Schwert | Schwerd |
| hell | häll | Hütte | Hüte |  | Sohn | Son | Brot | Brod |
| nett | nätt | Koffer | Kofer |  | Wohnung | Wonung | Blut | Blud |
| Schmerz | Schmärz | Gruppe | Grupe |  | Huhn | Hun | besorgt | besorgd |
| Stern | Stärn | Keller | Keler |  | Stuhl | Stul | breit | breid |
| Zettel | Zättel | Teller | Teler |  | Schuh | Schu | erstaunt | erstaund |

*Note*. ‡Items excluded

Table 4

*Orthographic Decision Items: Low Frequency Basewords and Pseudohomophones (PsH)*

| Baseword | PsH | Baseword | PsH |  | Baseword | PsH | Baseword | PsH |
| --- | --- | --- | --- | --- | --- | --- | --- | --- |
| Dämpfe | Dempfe | Bolzen | Bollzen |  | Folie | Fohlie | Tugend | Tugent |
| Tänzer | Tenzer | Bremse | Bremmse |  | Idol | Idohl | Magd | Magt |
| fädeln | fedeln | Tempel | Temmpel |  | Südpol | Südpohl | Pfund | Pfunt |
| Gefäß | Gefeß | Detail | Dettail |  | Koloss‡ | Kohloss‡ | Pfand | Pfant |
| Gebäck‡ | Gebeck‡ | Petze | Pettze |  | Signal | Signahl | Spind | Spint |
| Käufer | Keufer | Rotz | Rottz |  | Pate | Pahte | Fund | Funt |
| Läuse | Leuse | Tatze | Tattze |  | Taler | Tahler | Rind | Rint |
| Säbel | Sebel | Kritik | Krittik |  | Kram | Krahm | Elend | Elent |
| Krähe | Krehe | Witwe | Wittwe |  | Scham | Schahm | Rekord | Rekort |
| mähen | mehen | Bischof | Bischoff |  | Samen | Sahmen | Schlund | Schlunt |
| Heck | Häck | Hummel | Humel |  | Pfahl | Pfal | Anwalt | Anwald |
| wellig | wällig | Klemme | Kleme |  | Naht | Nat | bemüht | bemühd |
| Sensor | Sänsor | Gramm | Gram |  | Wahn | Wan | Diktat | Diktad |
| Ferkel | Färkel | Trümmer | Trümer |  | Sahne | Sane | Fakt | Fakd |
| Stempel | Stämpel | Motte | Mote |  | Kuhle | Kule | betont | betond |
| Fett | Fätt | Kutte | Kute |  | Lehm | Lem | gewagt | gewagd |
| Kurbel | Kurbäl | Kittel | Kitel |  | Mehl | Mel | Glut | Glud |
| felsig | fälsig | Waffel | Wafel |  | Lehne | Lene | Knast | Knasd |
| Ekel | Ekäl | Ziffer | Zifer |  | Floh | Flo | Quadrat | Quadrad |
| Benzin | Bänzin | Mappe | Mape |  | Lohn | Lon | Samt | Samd |

*Note*. ‡Items excluded
